# A Meta-Analysis of Experiments Linking Incubation Conditions with Subsequent Leg Weakness in Broiler Chickens

**DOI:** 10.1371/journal.pone.0102682

**Published:** 2014-07-23

**Authors:** Peter J. Groves, Wendy I. Muir

**Affiliations:** Faculty of Veterinary Science, The University of Sydney, Camden, NSW, Australia; Auburn University, United States of America

## Abstract

A series of incubation and broiler growth studies were conducted using one strain of broiler chicken (fast feathering dam line) observing incubation effects on femoral bone ash % at hatch and the ability of the bird to remain standing at 6 weeks of age (Latency-To-Lie). Egg shell temperatures during incubation were consistently recorded. Parsimonious models were developed across eight studies using stepwise multiple linear regression of egg shell temperatures over 3-day periods and both bone ash at hatch and Latency-To-Lie. A model for bone ash at hatch explained 70% of the variation in this factor and revealed an association with lower egg shell temperatures during days 4–6 and 13–15 and higher egg shell temperatures during days 16–18 of incubation. Bone ash at hatch and subsequent Latency-To-Lie were positively correlated (r = 0.57, P<0.05). A model described 66% of the variation Latency-To-Lie showing significant association of the interaction of femoral ash at hatch and lower average egg shell temperatures over the first 15 days of incubation. Lower egg shell temperature in the early to mid incubation process (days 1–15) and higher egg shell temperatures at a later stage (days 16–18) will both tend to delay the hatch time of incubating eggs. Incubation profiles that resulted in later hatching chicks produced birds which could remain standing for a longer time at 6 weeks of age. This supports a contention that the effects of incubation observed in many studies may in fact relate more to earlier hatching and longer sojourn of the hatched chick in the final stage incubator. The implication of these outcomes are that the optimum egg shell temperature during incubation for broiler leg strength development may be lower than that regarded as ideal (37.8°C) for maximum hatchability and chick growth.

## Introduction

The prevalence of observable leg abnormalities in commercial broiler chickens is generally reported as less than 3% [1], [2] however an abundance of broilers are affected in less obvious ways [3]. Affected individuals show modifications in motion, reduced ability to remain physically active and an increased incidence of lameness, all of which result in a reduced capacity to compete for food and water potentially leading to starvation and dehydration [4], [5]. Leg issues and gait abnormalities are regarded as the predominant causes of mortality and culling in broilers [3].

The etiology of leg weakness is complex and has been linked with multiple factors relating to genetics, bone development, pathology, hormonal control, nutrition, physical activity or factors related to environment or management [6]–[9]. Thorough understanding of all causative factors and their interactions are lacking and are not mutually exclusive as one or several can be associated with the incidence of leg weakness at any one time [10], [11].

In recent years there has been increased focus on variations in incubation conditions that may possibly contribute to leg problems in broiler chickens [3], . Ideal incubation temperatures are regarded as being between 37.5 to 38°C and 50 to 70% relative humidity [16], [20] with an overall “ideal” embryonic temperature target of 37.8°C [16], [17], [21]. Any deviations from this range have been incriminated with reduced locomotive integrity [3], [18], [19], [22]. Spraddle legs in broilers have been associated with high humidity during incubation [23], and cyclic overheating during the first 8 days of incubation has been implicated in the later incidence of Tibial Dyschondroplasia via an effect on growth plate hypoxia [13]. Other work has shown that pre-heating conditions of eggs prior to incubation could affect bone characteristics of chicks at hatch and the incidence of twisted legs as late as 40 days of age [24]. The latter authors also described effects on bone development and characteristics following early cool and/or late high temperature profiles and low oxygen tensions used during parts of the incubation process. Soft tissue effects have also been seen. In further experiments the same authors demonstrated an effect of an early low and later high incubation temperature profile in producing thinner gastrocnemius tendon fibres and differing collagen banding patterns during subsequent growth [19]. The temperatures used in all of these studies though were outside the normal realms of incubation practice (36°C and 39°C).

There is some argument over the exact mechanism of the observed effects on leg strength of incubation variations. A recent article concluded that most studies had failed to take into account the effect of hatching time and that the time that hatched chicks spend in the final stage incubator is actually the major determinant of the leg problems which eventuate [25]. This study has been criticized however for the use of a single low temperature (36°C) during the final days of incubation [1].

Bone mineralization and development starts during early embryo development [26] so it is feasible that leg abnormalities may originate during the incubation period. It has been established that embryonic temperature is more important than the air temperature recorded by the incubator [27] as these can vary markedly. It has also been demonstrated that egg shell temperature during incubation (EST) is a close measure of actual embryonic temperature [27], [28] and it has been recommended that all experiments investigating incubation effects should record and report EST [28].

While broiler bone quality has been shown to be impacted by less than optimal incubation conditions, limited research has been conducted to investigate whether incubation conditions within standard industry practice ranges could affect leg integrity. A series of studies in our laboratory [29] attempted to associate measureable effects on the leg integrity of a fast feathering dam parent line (L12) as a result of variations in incubation profiles within the recommended range of incubation conditions. Results from various profiles gave inconsistent results but did indicate that incubation variations could produce variations in bone mineralization at hatch, demonstrated by the lower femoral bone ash levels and changes in levels of serum calcium and phosphorus of hatchlings (unpublished data). Chicks exposed to variations of incubation profile also demonstrated changes in their willingness to remain standing, as determined by the latency-to-lie (LTL) test at 5–6 weeks of age. Subsequent experiments with increased egg shell temperatures during incubation (EST) were inconsistent in provoking measurable bone or leg weakness. To better understand the patterns that may have been hidden within a substantial incubation data set, a multiple regression analysis [30] was conducted on the combined output of eight incubation experiments.

## Materials and Methods

### Animal Ethics

All experimental procedures were approved by the University of Sydney Animal Ethics Committee (protocol approval numbers N00/9-2009/1/5145 and N00/2-2011/2/5461) and were conducted under strict compliance with the Australian Code of Practice for the Care and Use of Animals for Scientific Purposes as prepared by the National Health and Medical Research Council, 2013.

### Experimental designs and procedures

Eight incubation experiments with varied temperature profiles from 0 to 18 days of incubation were evaluated in this analysis. In all, a total of 26 separate incubations were conducted throughout the trials. The variations measured in each incubation are shown in Table S1 and Table S2 in File S1.

In all experiments, after the first 18 days of incubation, all eggs were transferred to a single incubator for hatching at 21 days and 12 hours (516 hours). The hatching period incubator was set at 37.4°C air temperature at egg transfer (18 days of incubation) and this was progressively decreased to 36.9°C by 21 days and 12 hours of incubation with relative humidity starting at 60% and rising to 65% over the same time period.

At hatch, a random sample of chicks (n = 40) from each incubation group were selected, had blood samples collected, were weighed and their length from beak tip to toe nail [31] was measured. The sample chicks were then humanely euthanized and their yolk weight determined, yolk contents stored and the right femur was collected for bone ash analysis. All eggs which failed to hatch were examined for stage of failure.

In all experiments where chicks were grown out, remaining hatched chicks were housed in floor pens in an environmentally controlled building until 38–42 days of age. Commercially available broiler starter and finisher rations were obtained and fed *ad libitum* to all birds. Birds from each incubation treatment were identified by toe marking and a numbered wing badge at day of hatch. Birds from each experimental group were mixed in each floor pen used, so that environmental experience for each group was as close to identical as possible.

### Incubators

Experiments 1, 2 and 3 used two Aussieset incubators (Bellsouth Pty Limited, Victoria, Australia). Maximum capacity of each of these incubators is 2000 eggs, with digital temperature control, humidity provided by sprays, automated turning and forced ventilation. These incubators proved difficult to control to the desired precision and exhibited some variation from the intended experimental design during incubation.

Experiments 4 to 8 used four smaller 288-egg-capacity incubators (E2A - Multiquip Pty Limited, Austral, New South Wales, Australia). These incubators have forced ventilation and evaporative humidity provision, digital temperature control and automated turning.

A single Aussieset incubator was used for the hatching period in all experiments with eggs from all setter incubators randomised throughout.

### Incubation measurements

In each experiment, temperature and humidity data loggers (Zenith AZ8829, supplied by Bacto Laboratories, Liverpool, NSW, Australia) were placed in each machine in close proximity to the eggs. Data from these were downloaded to a computer following the completion of incubation. Egg shell temperatures (EST) were measured in all experiments except experiment 5. In experiments 1 through 4 and 6, this was done using a hand held infrared thermometer (Exergen DX501 Precision IR Thermometer) placed at the equator of at least ten eggs in each machine. In experiments 7 and 8, Remote Intelligent Multisensors (TSIC 716 Advanced Sensor Technology – supplied by Netic Pty Limited, Ryde, NSW, Australia) were attached to four egg fillers in each incubator and contact with the equator of the egg was maintained with thermal paste (Silicon heat transfer compound, Unick Chemical Corp.). The sensors were connected to a remote physical monitor (Uptime Devices, Ryde, NSW, Australia) and to a notebook computer where a software program (Net Sensor Man, Netic Pty Limited) recorded temperatures continuously at 1 minute intervals from days 1 to 18 of incubation. The relationships between EST and air temperatures measured in the incubators is shown in Table S3 in File S1.

Digital and thermometer readouts (temperature and humidity) on each incubator were recorded at multiple times daily in each experiment.

Chicks were not grown out in experiment 2.

### Serum calcium and phosphorus analysis

Assays for serum calcium and phosphorus were conducted by the University of Sydney Veterinary Teaching Hospital, Camden. Total serum calcium was estimated using the metallochromagen Arsenazo III reagent (Catalogue No. TR29226, Thermo Fisher Scientific Inc., Middletown, VA, USA) which forms a coloured chromophore with Ca ions at pH 6.75, measured at 650 nm. Serum inorganic phosphorus concentration was measured using an inorganic P reagent (direct UV method without reduction) producing unreduced phosphomolybdate and measured at 340 nm (Catalogue No. TR 30026, Thermo Fisher Scientific Inc., Middletown, VA, USA). Colorimetric assessments were conducted using a Konelab 20 XTi (Thermo Electron) clinical chemistry analyser.

### Bone ash analysis

Femur samples were frozen subsequent to collection. The bones were cleaned of any adhering tissue by placing them in boiling water. The bones were then weighed before being placed in to a drying oven at 105°C for 24 hours. Dry bone weights were than taken before the bone was placed in to a muffle furnace set at 200°C and temperatures were increased by 100°C increments with corresponding time pause until 600°C. After 8 hours, samples were removed from the furnace, allowed to cool in a dessicator and the weight of the remaining bone ash was recorded.

### Latency to Lie

Between 38 and 42 days across these experiments, a sample of birds were selected and subjected to a modified Latency-To-Lie (LTL) test. The method used was based on an established procedure [32], [33]. Briefly, at least 30 randomly selected birds per treatment were placed in a tub containing a depth of approximately 3 cm of water at 31–33°C and timed for length of their ability to remain standing, up to a maximum of 5 minutes. As the test was terminated at 5 minutes it could not be determined for how long some birds may have stood, hence the mean value was not meaningful. Hence median standing time was used for comparison across experiments.

### Statistical Analyses

All statistical analyses were conducted using the computerised statistics package JMP ver 9.0.0 software (SAS Institute Inc., Cary, NC, USA, 2010).

Data which was consistently recorded between the incubation studies included daily egg shell temperatures (EST) measured by either method described above, relative humidity percentage (RH%) during incubation, percentage late deaths during incubation (embryos >8 cm in length), chick hatch weights, chick length at hatch, femoral bone ash (BA) at hatch, serum calcium and phosphorus at hatch, chick weight at 7 days of age and median latency-to-lie (LTL) at 38 to 42 days of age. EST was averaged over 3-day intervals during the first 18 days’ incubation (i.e. days 1–3, 4–6, 7–9, 10–12, 13–15 and 16–18 days respectively). RH% varied substantially over each day and was averaged across days 1 to 9 and 10 to 18 of incubation.

A meta-analysis is regarded as a powerful method for pooled analyses, particularly where the variables measured are consistent between studies [30]. A meta-analysis which utilizes the individual data from a group of experiments can improve the understanding of the main effects but can also examine interactions between factors and groups and can overcome confounding [30]. Meta-regression may be appropriately used where the putative effect-modifying factors are quantitative [30].

The two parameters to be used as dependent variables (outcomes) chosen were femoral bone ash % at hatch (BA) and median latency-to-lie (LTL) at 38–42 days of age as these relate directly to leg weakness.

The assumptions for a valid linear regression are that the variables are related linearly and that the errors are independent, normally distributed with zero mean and have a constant variance. The multiple regression analysis procedure followed a recommended approach [34]. Briefly, to select variables that could be logically incorporated into a statistical model, all parameters were plotted against each other and assessed for linear graphical association. Descriptive statistics for these factors are shown in Table 1. Correlation coefficients between all variables were calculated (pairwise Pearson correlation coefficients if associations were linear or Spearman Rank correlation coefficients if non-linear - Table 2) and the probability that the associations seen may have been due to chance were calculated.

**Table 1:** Descriptive statistics for measured factors across eight broiler egg incubation experiments made up of 26 individual incubations.

	Units	Valid N	Mean	Minimum	Maximum	StandardDeviation	SE^a^	Skewness	Kurtosis	Shapiro-WilkW test^j^, P =
Hatch weight^b^	gm	22	42.74	37.38	45.97	2.84	0.61	−0.08	−0.89	0.01
Hatch length^c^	cm	22	18.4	17.65	19.42	0.55	0.12	0.37	−1.34	0.03
Hatch femur ash^d^	%	26	26.85	23.36	29.20	1.86	0.36	−0.50	−0.84	0.0504
Hatch serum Ca	mmol/litre	26	2.35	1.93	2.64	0.19	0.04	−0.61	−0.20	0.23
Hatch serum P	mmol/litre	26	1.13	0.87	2.32	0.37	0.07	2.77	7.41	0.00
Weight^e^ 7 days	gm	24	144.7	127	172	11.37	2.32	0.59	−0.02	0.32
Median LTL^f^ d42	seconds	24	124.9	65	226	43.10	8.80	0.64	0.04	0.26
Late dead^g^	% of fertile eggs	24	12.02	4.76	36.31	7.64	1.56	1.71	3.43	0.00
RH 1–9^h^	%	26	53.4	36.7	66.4	6.70	1.31	−0.86	1.26	0.10
RH 10–18^h^	%	26	52.8	37.6	66.1	6.84	1.34	−0.80	0.52	0.02
EST 1–3^i^	°C	22	37.5	36.3	38.2	0.49	0.10	−0.36	−0.17	0.44
EST 4–6^i^	°C	22	37.5	36.0	38.4	0.60	0.13	−0.91	0.68	0.11
EST 7–9^i^	°C	22	37.5	35.5	38.5	0.73	0.16	−1.01	1.19	0.16
EST 10–12^i^	°C	22	37.6	36.3	38.6	0.58	0.12	−0.09	0.01	0.70
EST 13–15^i^	°C	22	37.8	36.8	38.9	0.52	0.11	0.29	0.00	0.96
EST 16–18^i^	°C	22	38.0	37.1	39.1	0.49	0.10	0.66	0.66	0.45

a Standard error of the mean.

b Weight of chicks at hatch.

c Length of chick at hatch (31).

d Bone ash % of femur of chicks at hatch.

e Weight of chicks at 7 days of age.

f Median value of Latency To Lie test conducted between 38 and 42 days of age.

g Feathered embryos >8 cm in length (31) which died during incubation as a percentage of fertile eggs sett.

h Percent Relative Humidity within each incubator measured over 1 to 9 or 10 to 18 days of incubation.

I Mean Egg Shell Temperature over the days of incubation specified.

j Shapiro−Wilk W test assesses whether a sample’s distribution differs significantly from Normal.

**Table 2:** Correlation coefficients (pairwise r) between parameters measured over eight incubation experiments.

Correlationcoefficient	HatchWeight^a^	HatchLength^a^	Hatchfemur ash %^b^	Hatchserum Ca	Hatchserum P^a^	MedianLTL^c^	EST^d^1–3	EST^d^4–6	EST^d^7–9	EST^d^10–12	EST^d^13–15	EST^d^16–18	EST^d^1–15	RH%^e^1–9
Hatch Length	**0.72** *													
Hatch femur ash %	**0.51** *	0.25												
Hatch serum Ca	−0.16	0.26	0.02											
Hatch serum P^a^	0.18	0.28	−0.24	−0.35										
Median LTL	0.26	0.05	**0.57** *	0.05	−0.37									
EST1–3	−0.30	−0.16	−**0.46** *	−0.07	0.68*	−**0.62** *								
EST 4–6	−**0.59** *	−0.16	−**0.61** *	0.24	0.49*	−**0.55** *	**0.80** *							
EST 7–9	−**0.59** *	−0.26	−**0.56** *	0.28	0.40	−**0.65** *	**0.75** *	**0.93** *						
EST 10–12	−**0.60** *	−0.36	−**0.51** *	0.40	0.22	−**0.56** *	**0.56** *	**0.72** *	**0.85** *					
EST13–15	−**0.57** *	−0.45	−**0.39** *	0.35	0.15	−**0.47** *	0.40	**0.47** *	**0.63** *	**0.91** *				
EST 16–18	0.18	0.15	0.19	0.40	−0.19	−0.06	0.03	0.07	0.26	**0.57** *	**0.75** *			
EST 1–15	−**0.56** *	−0.18	−**0.59** *	0.27	0.11	−**0.69** *	**0.79** *	**0.90** *	**0.96** *	**0.93** *	**0.77** *	0.38		
RH%^f^ 1–9	0.19	0.10	0.05	0.06	−0.26	**0.46** *	−0.33	−0.23	−0.28	−0.38	−0.40	−0.20	−0.35	
RH%^f^10–18^a^	0.09	0.25	0.00	0.34	−0.30	0.33	−0.30	−0.09	−0.08	−0.13	−0.18	−0.05	−0.14	**0.86** *

*Coefficient differs significantly from zero (P<0.05).

a Spearman Rank correlation coefficients calculated for these variables; all other values are Pearson correlation coefficients.

b Bone ash % of femur of chicks at hatch.

c Median value of Latency To Lie test conducted between 38 and 42 days of age.

d Mean Egg Shell Temperature over the days of incubation specified.

e Percent Relative Humidity within each incubator measured over 1 to 9 or 10 to 18 days of incubation.

A multiple regression analysis was then performed for each selected dependent variable against other variables which had shown significant (P<0.05) correlation with them and that could be considered to be putative determinants of the outcome. Therefore, the multiple regression model for BA as the dependent variable evaluated all EST periods and the relative humidity variables (RH), while those for LTL as dependent variable included chick weight at hatch, BA, all EST periods and RH over days 1–9 of incubation.

The objective of these analyses was to determine the most parsimonious models possible to explain variation in the dependent variables. Initially, all selected variables were run in a multiple regression and evaluated for their contribution to the overall model. A forward stepwise regression was then run on this model using the minimization of BIC (Schwartz’s Bayesian Information Criteria) stopping rule to eliminate redundant variables. Then all possible interactions of the retained variables were added to the model and a further forward stepwise regression was conducted to produce a final best fit model. If interaction terms were significant, the individual variables for that interaction were retained in the model even if they were not significant on their own [34].

Following development of the final best fit model, several diagnostic procedures were carried out to evaluate if statistical assumptions for this analysis were met. Actual versus predicted values were graphed and examined for agreement with a linear relationship, with the 95% confidence limits for the line of best fit not including the baseline model (null hypothesis) and to evaluate the presence of outliers or bias. Residual versus predicted values were graphed and assessed for absence of appearance of any patterns in the data. Then a residuals by row plot was evaluated to assess the independence of the data or the presence of autocorrelation (the Durbin-Watson statistic was calculated).

## Results

Table 1 shows descriptive statistics for all variables considered for entry into the analysis. The objective of the analyses was to identify possible variables which could predict or affect the outcome (dependent) variables of femoral bone ash % at hatch (BA) and median Latency-to-lie at 38–42 days of age (LTL). Table 2 is a triangular matrix of pairwise correlation coefficients between each variable. Where data were not normally distributed (as denoted by a significant Shapiro-Wilk W, P<0.05, in Table 1), Spearman Rank coefficients were calculated instead of Pearson correlation coefficients. Only variables which occurred prior to the occurrence of the outcome variable could be included and only if they were practically able to be varied to affect the outcome variable.

EST period means were significantly correlated to each other, which would be expected as incubator temperatures were varied to minimal extents within each experiment.

### Femoral bone ash at hatch (BA)

For BA, variables meeting the criteria to be selected for inclusion in the multiple regression analysis included chick hatch weight (CW), and egg shell temperatures (EST) averaged over days 1 to 3 (EST 1–3), 4 to 6 (EST 4–6), 7 to 9 (EST 7–9), 10 to 12 (EST 10–12) and 13 to 15 (EST 13–15) (Table 3). CW however was not normally distributed and showed a bi-phasic distribution. CW was also strongly negatively correlated to EST terms over days 4–6, 7–9, 10–12 and 13–15 (Table 2). CW is a factor of initial egg weight and time that the chick has been out of the egg (as weight decreases due to weight and moisture loss after hatching without access to feed or water). Time of hatching is determined by incubation profile [25], [27] and hence inclusion of this term in the regression could lead to autocorrelation and distortion of outcomes. In terms of putative predictive variables that could actually be manipulated, this left only the EST's as selected contributory variables. Hence only the EST terms mentioned above were included in the analysis model for BA.

**Table 3:** Final stepwise^a^ multiple regression model for femoral ash % at hatch (BA).

Parameter	Estimate (β)	SE^b^	t-Ratio^c^	Prob>|t|
Intercept	56.56	24.14	2.34	0.0324
EST 4–6	−1.37	0.63	−2.19	0.0434
EST 13–15	−2.56	1.02	−2.52	0.0229
(EST 4–6)×(EST 13–15)	−2.28	1.10	−2.07	0.0547
EST 16–18	3.13	0.89	3.53	0.0028
(EST13–15)×(EST 16–18)	0.52	0.71	0.73	0.4784
**Summary of fit**				
n = 22	R^2^ = 0.77	AICc = 78.23	Durbin-Watson statistic = 2.303	
RMSE = 1.02	Adjusted R^2^ = 0.70	BIC = 77.87	Autocorrelation = −0.1639	
	Cp = 5.03, p = 6		Prob <DW = 0.70	

a Forward stepwise multiple regression using the minimum BIC stopping method.

b standard error of the mean.

c Student’s t-test ratio.

The EST terms that were selected and their full factorial interaction terms were subjected to forward stepwise multiple regression with BA as the dependent variable. Results are shown in Table 3. The final best fit model included the EST variables for days 4 to 6, 13 to 15 and 16 to 18 of incubation and the interaction term between EST 4–6 and EST 13–15 and also the interaction term between EST 13–15 and EST 16–18. The effects of the individual EST terms were significant (P<0.05). The inclusion of the interaction terms improved the overall fit of the model but these terms were not significant alone (Table 3). There were significant negative associations between BA and EST at 4–6 and 13–15 days, while there was a significant positive association between BA and EST 16–18.

Hence the best deducible predictors of femoral bone ash % at hatch as a result of incubation differences was a negative correlation with EST earlier in incubation (i.e. lower EST in early incubation is associated with higher BA at hatch) and a positive relationship with late incubation temperatures (EST 16–18).

### Latency-to-lie at 6 weeks (LTL)

For LTL, variables meeting the criteria to be selected for inclusion in the multiple regression analysis included all EST’s from 1 to 15 days, percent relative humidity between days 1 to 9 (RH 1–9) and hatch femoral bone ash % (BA). Including all these variables plus their second degree interactions produced a model which failed to converge, producing biased outcomes. As all EST values were strongly correlated to each other (Table 2), to overcome this problem EST over days 1 to 15 were collapsed into a single variable (EST 1–15). BA and RH 1–9 and all interactions between these variables were also included in this reduced model to create a more parsimonious regression model. Adopting a forward stepwise regression analysis selected an interaction between EST 1–15 and BA, the individual EST 1–15 and BA terms and RH 1–9 remaining as significant factors in the model (Table 4). This equation produced an adjusted R^2^ for the model to 0.66 and an RMSE of 26.19. A backward stepwise regression yielded an identical outcome. There were no apparent patterns in residual by predicted nor residual by row plots. The Durbin-Watson statistic for this equation showed no significant autocorrelation (P = 0.90).

**Table 4:** Final stepwise^a^ multiple regression model for Latency-To-Lie at 38–42 days of age (LTL).

Parameter	Estimate (β)	SE^b^	t-ratio^c^	Prob >|t|
Intercept	743.3	946.9	0.78	0.4447
(BA)×(EST 1–15)	−31.67	10.42	−3.04	0.0083
EST 1–15	−26.75	21.98	−1.22	0.2423
BA	9.04	4.84	1.87	0.0813
RH 1–9	2.29	1.15	1.98	0.0661
**Summary of fit**	R^2^ = 0.73	AICc = 200.1		
n = 20	Adjusted R^2^ = 0.66	BIC = 199.6	Auotcorrelation = −0.36	
RMSE = 26.19	Cp = 4.37, p = 5	Durbin-Watson = 2.66	Prob <DW = 0.90	

a Forward stepwise multiple regression using the minimum BIC stopping method.

b standard error of the mean.

c Student’s t-test ratio.

Hence LTL had a negative relationship with EST over the first 15 days of incubation and a positive response to higher BA and RH 1–9. In other words lower EST from days 1 through 15 of incubation with higher relative humidity in the first 9 days of incubation and higher hatch bone ash at hatch were associated with longer latency-to-lie results at 5–6 weeks of age.

## Discussion

Over the course of eight incubation experiments looking at possible effects of variations in incubation conditions on subsequent skeletal integrity in a fast feathering broiler chicken parent strain, the two most meaningful parameters evaluated which estimated leg strength were found to be femoral bone ash % at hatch and latency-to-lie at 5–6 weeks of age. Attempts were made in this course of experiments to deleteriously affect broiler leg strength by increasing incubation temperatures (as measured by egg shell temperature) at various stages of incubation as suggested by much of the literature [3], [10], [13], [18], [35]. This proved difficult to consistently achieve and was hampered by inherent variations in temperature control with the incubators used in these experiments. The meta-regression of all eight experiments, using consistent measurements and the same strain of bird within each, provided a significant indication that bone mineralization at hatching could be improved by a lower temperature of the egg shell during the first 15 days of incubation, but not over days 16–18 of incubation. It also indicated that bone ash at hatch and later locomotory ability of the chicken could be positively linked and that lower incubation temperatures over the first 15 days of incubation and higher relative humidity over the first 9 days of incubation were also associated with the birds’ ability to stand for longer periods by 5–6 weeks of age.

All of the identified incubation variations that were found to be associated with improved leg strength would also function to slow down embryonic development. During early embryogenesis, the rate of development is essentially anaerobic and enzyme driven and hence will be accelerated by higher temperature [27], [28], [36]. However once the chorioallantoic membrane is established by day 7–9, the embryo will draw oxygen through the shell and aerobic respiration begins. As the embryo grows, oxygen becomes the limiting factor for continued development after days 15–16. An increase in embryonic temperature at this later phase of incubation will slow development [12], [35]. The timings explained here fit well with the observed associations from this analysis.

The egg shell temperature which is regarded as “ideal” to maximize hatchability is 37.8°C [16], [20], [27]. It has been observed by some that this ideal for hatchability may not necessarily be the optimum for broiler growth [27], [37]. One study [25] concluded that many of the reported experiments looking at leg weakness associated with incubation failed to take into account the time of chick hatching and the subsequent sojourn of the hatched chick in the hatcher, and that it was this latter factor that resulted in the development of subsequent leg problems. Our findings from this analysis would agree with the latter hypothesis. In fact, in the experiments where leg strength could demonstrably be shown to be better, the chicks were observed to hatch late and were still markedly wet at take off from the hatcher baskets.

While many studies have evaluated the deleterious effects of deviation from the ideal incubation temperature for hatchability has on broiler leg strength, the current analysis points towards some variations in accepted incubation techniques that may improve broiler locomotory ability. It must be noted that the observations here are relevant only to the fast feathering female parent line of one major broiler strain. Differences in response between broiler strains to the same incubation conditions have been documented [38] with the Cobb broiler strain known to develop faster, generate more heat and hatch earlier than the Ross broiler strain under the same conditions. Perhaps a portion of the differences in leg weakness characteristics observed between these breeds [6] in the field may be due to differing responses to incubation conditions. This deserves more thorough investigation. From the regression equations generated it may be possible to design experimental incubation profiles that may predict variations in bone ash at hatch and later leg weakness.

## Supporting Information

File S1 **Supporting tables.**
(DOCX)

Checklist S1 **PRISMA checklist.**
(DOC)

Flowchart S1 **PRISMA 2009 flow diagram.**
(DOC)
