# Supplementary material for: A Meta-Analysis of Experiments Linking Incubation Conditions with Subsequent Leg Weakness in Broiler Chickens
Source: PLoS One. 2014 Jul 23;9(7):e102682. doi: 10.1371/journal.pone.0102682 (PMC4108365; doi:10.1371/journal.pone.0102682)
Supplement: File S1 — Supporting tables. (DOCX) [file pone.0102682.s001.docx]

Table S1. Incubation profile recordings over all experiments -means over each period.

| Experiment Number | Incubation number | EST^a^ 1-3 | EST^a^ 4-6 | EST^a^ 7-9 | EST^a^ 10-12 | | EST^a^ 13-15 | EST^a^ 16-18 | | RH%^b^ 1-9 | RH%^b^ 10-18 |
| --- | --- | --- | --- | --- | --- | --- | --- | --- | --- | --- | --- |
| 1 | 1 | 38.0 | 37.5 | 37.7 | 37.9 | 37.9 | | 37.6 | 53.1 | | 53.4 |
|  | 2 | 37.4 | 37.8 | 37.3 | 36.9 | 36.8 | | 37.1 | 57.4 | | 55.2 |
| 2 | 3 | 36.3 | 36.0 | 35.4 | 36.3 | 37.2 | | 37.3 | 53.3 | | 53.3 |
|  | 4 | 38.2 | 38.4 | 38.2 | 37.9 | 38.0 | | 38.3 | 55.9 | | 56.2 |
| 3 | 5 | 37.9 | 37.7 | 37.4 | 37.7 | 38.2 | | 38.1 | 53.0 | | 52.9 |
|  | 6 | 37.6 | 37.7 | 37.7 | 37.2 | 37.2 | | 37.4 | 53.0 | | 53.0 |
| 4 | 7 | 37.4 | 36.7 | 36.4 | 37.3 | 37.8 | | 38.4 | 51.9 | | 43.3 |
|  | 8 | 37.4 | 36.9 | 37.2 | 37.1 | 37.5 | | 37.9 | 54.4 | | 41.2 |
|  | 9 | 36.9 | 36.7 | 36.8 | 37.4 | 37.7 | | 38.3 | 59.7 | | 59.6 |
| 5 | 10 |  |  |  |  |  | |  | 36.7 | | 38.5 |
|  | 11 |  | Not recorded | |  |  | |  | 50.6 | | 54.2 |
|  | 12 |  |  |  |  |  | |  | 44.7 | | 44.2 |
|  | 13 |  |  |  |  |  | |  | 59.5 | | 57.9 |
| 6 | 14 | 36.8 | 37.0 | 36.5 | 37.3 | 37.6 | | 38.2 | 55.7 | | 54.1 |
|  | 15 | 37.4 | 37.5 | 37.4 | 37.6 | 37.6 | | 38.1 | 58.0 | | 60.7 |
|  | 16 | 37.0 | 37.2 | 36.8 | 37.0 | 37.3 | | 37.9 | 66.4 | | 66.1 |
|  | 17 | 37.1 | 37.2 | 37.1 | 37.0 | 37.2 | | 37.8 | 55.8 | | 55.5 |
| 7 | 18 | 37.1 | 37.6 | 37.7 | 37.8 | 37.9 | | 37.8 | 58.4 | | 56.3 |
|  | 19 | 37.2 | 37.7 | 37.7 | 37.8 | 37.9 | | 37.8 | 57.5 | | 56.6 |
|  | 20 | 38.1 | 38.1 | 38.1 | 38.0 | 38.1 | | 37.7 | 61.9 | | 58.6 |
|  | 21 | 38.1 | 38.2 | 38.1 | 38.1 | 38.1 | | 37.7 | 38.2 | | 37.6 |
|  | 22 | 37.5 | 38.2 | 38.3 | 38.6 | 38.4 | | 38.1 | 55.5 | | 53.9 |
| 8 | 23 | 37.4 | 37.7 | 37.9 | 37.9 | 37.9 | | 38.1 | 52.5 | | 55.8 |
|  | 24 | 37.2 | 37.7 | 38.1 | 38.5 | 38.7 | | 39.0 | 45.0 | | 48.6 |
|  | 25 | 37.9 | 38.1 | 37.6 | 37.5 | 37.4 | | 37.3 | 51.2 | | 54.2 |
|  | 26 | 37.9 | 38.2 | 38.5 | 38.6 | 38.9 | | 39.1 | 49.1 | | 51.9 |

^a^ Mean Egg Shell Temperatures (^o^C) over the days of incubation specified.

^b^ Mean Relative Humidity % over the days of incubation specified

Table S2. Measurements at hatch and during grow out in all eight experiments

| Experiment Number | Incubation number | Chick Weight  gm | Chick Length  cm | | Hatch bone ash % | | Hatch serum Ca  Mmol/l | Hatch serum P  Mmol/l | Weight d7  gm | | Weight d14  gm | Median LTL d42  secs | | Hatch % | Late dead%^\a^ |
| --- | --- | --- | --- | --- | --- | --- | --- | --- | --- | --- | --- | --- | --- | --- | --- |
| 1 | 1 |  |  | | 25.3 | | 2.1 | 2.3 | 138 | 400 | | 94 | 77.2 | | 4.9 |
|  | 2 |  |  | | 26.9 | | 1.93 | 2.32 | 128 | 381 | | 149 | 74.5 | | 7.7 |
| 2 | 3 |  |  | | 26.3 | | 1.97 | 1 | Not grown out | | | | | | |
|  | 4 |  |  | | 24.4 | | 2.2 | 1.12 |  |  |  |  |  |  |  |
| 3 | 5 | 43.70 | 18.29 | | 27.5 | | 2.28 | 1.35 | 172 | 413 | | 91 | 73.1 | | 5.3 |
|  | 6 | 45.11 | 19.13 | | 28.3 | | 2.17 | 1.22 | 162 | 393 | | 130 | 76.8 | | 5.3 |
| 4 | 7 | 44.89 | 17.98 | | 28.9 | | 2.2 | 0.93 | 127 | 337 | | 142 | 75.1 | | 8.8 |
|  | 8 | 45.97 | 18.28 | | 28.6 | | 2.1 | 1.06 | 131 | 329 | | 156 | 68.8 | | 11.8 |
|  | 9 | 45.91 | 18.12 | | 29.1 | | 2.33 | 0.97 | 132 | 332 | | 148 | 79.8 | | 5 |
| 5 | 10 | 41.39 | 17.85 | | 29.1 | | 2.43 | 0.98 | 147 | 393 | | 110 | 71.87 | | 12.85 |
|  | 11 | 43.49 | 17.98 | 29.2 | | 2.34 | | | 0.94 | 159 | | 412 | 183 | | 73.61 |
|  | 12 | 39.52 | 18.09 | | 27.4 | | 2.27 | 0.93 | 160 | 408 | | 122 | 70.49 | | 13.54 |
|  | 13 | 43.05 | 18.12 | | 27.3 | | 2.36 | 0.87 | 155 | 398 | | 147 | 75.69 | | 9.72 |
| 6 | 14 | 44.22 | 19.02 | | 28.2 | | 2.6 | 0.91 | 153 | 381 | | 209 | 75.15 | | 10.41 |
|  | 15 | 44.13 | 19.42 | | 29 | | 2.64 | 1.03 | 153 | 389 | | 130 | 68.62 | | 17.63 |
|  | 16 | 45.07 | 18.94 | | 28.7 | | 2.64 | 0.96 | 150 | 373 | | 226 | 79.41 | | 6.29 |
|  | 17 | 43.39 | 18.46 | | 27.2 | | 2.55 | 0.95 | 146 | 376 | | 117 | 74.16 | | 6.46 |
| 7 | 18 | 39.13 | 17.69 | | 26.63 | | 2.49 | 0.89 | 142 |  | | 131 | 82.14 | | 4.76 |
|  | 19 | 39.45 | 17.93 | | 23.72 | | 2.46 | 0.87 | 139 |  | | 70 | 77.98 | | 10.12 |
|  | 20 | 38.35 | 17.94 | | 24.26 | | 2.4 | 1.09 | 142 |  | | 85 | 58.13 | | 26.19 |
|  | 21 | 37.38 | 17.65 | | 23.36 | | 2.44 | 1.22 | 140 |  | | 68 | 75 | | 16.67 |
|  | 22 | 37.73 | 17.70 | | 23.59 | | 2.43 | 0.97 | 140 |  | | 159 | 80.09 | | 6.48 |
| 8 | 23 | 45.85 | 19.04 | | 26.3 | | 2.45 | 1.06 | 138 | 375 | | 80 | 69.46 | | 15.26 |
|  | 24 | 44.21 | 18.93 | | 26.8 | | 2.47 | 1.21 | 138 | 368 | | 65 | 61.84 | | 21.73 |
|  | 25 | 45.11 | 19.02 | | 25.6 | | 2.44 | 1.02 | 141 | 379 | | 87 | 75.83 | | 15.13 |
|  | 26 | 43.18 | 18.85 | | 26.4 | | 2.5 | 1.16 | 139 | 377 | | 98 | 48.76 | | 36.31 |

^\a^ - dead in shell embryos >8cm length from beak to toe nail insertion.

Table S3. Pearson Correlation coefficients (**r**) between incubator and egg shell temperatures

| Temperature days^\a^ | IAT 1-3 | IAT 4-6 | IAT 7-9 | IAT 10-12 | IAT 13-15 | IAT 16-18 |
| --- | --- | --- | --- | --- | --- | --- |
| EST 1-3 | 0.58* | 0.71* | 0.74* | 0.64* | 0.57* | 0.37 |
| EST 4-6 | 0.53* | 0.83* | 0.94* | 0.83* | 0.61* | 0.33 |
| EST 7-9 | 0.49* | 0.76* | 0.93* | 0.87* | 0.71* | 0.38 |
| EST 10-12 | 0.38 | 0.63* | 0.80* | 0.87* | 0.80* | 0.47* |
| EST 13-15 | 0.25 | 0.44* | 0.57* | 0.73* | 0.80* | 0.60* |
| EST 16-18 | 0.07 | 0.08 | 0.10 | 0.22 | 0.37 | 0.33 |

^\a^ Incubator Air Temperature (IAT) and Egg Shell Temperature (EST) averaged over days of incubation as specified by the following numbers.

* Correlation coefficient differs significantly from zero (P<0.05).
