# Supplementary material for: A Meta-Analysis of Experiments Linking Incubation Conditions with Subsequent Leg Weakness in Broiler Chickens
Source: PLoS One. 2014 Jul 23;9(7):e102682. doi: 10.1371/journal.pone.0102682 (PMC4108365; doi:10.1371/journal.pone.0102682)
Supplement: Flowchart S1 — PRISMA 2009 flow diagram. (DOC) [file pone.0102682.s003.doc]

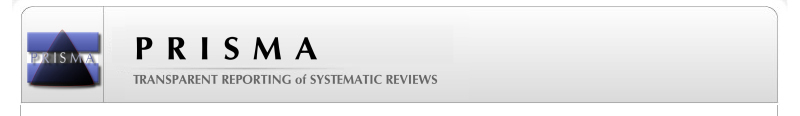
**PRISMA 2009 Flow Diagram**

**Screening**

**Included**

**Eligibility**

**Identification**

Records identified through database searching
(n = 0 )

Additional records identified through other sources
(n = 8 )

Records after duplicates removed
(n = 8 )

Records screened
(n = 8 )

Records excluded
(n = 0 )

Full-text articles assessed for eligibility
(n = )

Full-text articles excluded, with reasons
(n = )

Studies included in qualitative synthesis
(n = 8 )

Studies included in quantitative synthesis (meta-analysis)
(n = 8 )

**THIS IS NOT AN ARTICLE EXRACTION META-ANALYSIS**
